# Management of Adrenal Deficiency and Shock in a Patient With Polyglandular Autoimmune Syndrome Type II

**DOI:** 10.7759/cureus.41440

**Published:** 2023-07-06

**Authors:** Rebekah Lantz, Waseem Naboulsi, Sarah Yu, Maher Al-Samkari

**Affiliations:** 1 Hospitalist, Miami Valley Hospital, Dayton, USA; 2 General Medicine, Wright State University Boonshoft School of Medicine, Dayton, USA; 3 Hospital Medicine, Endocrinology, Premier Health Network, Dayton, USA

**Keywords:** myxedema crisis, diabetic ketoacidosis (dka), adrenal shock, endocrinologist and diabetologist, hospitalist medicine, autoimmune adrenal insufficiency, hypothyroid, type 1 diabetes mellitus (t1dm), autoimmune polyglandular syndrome type ii, endocrine disorders

## Abstract

Polyglandular autoimmune syndrome (PAS) is a rare disorder characterized by the autoimmune destruction of multiple endocrine glands. Type II PAS is the most common of the PAS subtypes and is characterized by Addison’s disease, autoimmune thyroid disease, and type I diabetes mellitus. Disease manifestations are predominantly seen in young adulthood with an emerging endocrine disorder; however, a host of other autoimmune conditions can also be present before endocrine organ dysfunction. Due to the complex nature of presentation and management, an important consideration in patient care involves a multidisciplinary team with the addition of an endocrinologist.

A 21-year-old African American woman with a medical history of PAS-II presented during three hospitalizations with adrenal crisis, diabetic ketoacidosis (DKA), and myxedema. The common theme across admissions entails a spectrum of adrenal dysfunction, including shock, as well as glucose and thyroid abnormalities.

During her first hospitalization, the patient presented with hypotension, hyperglycemia, and hypothyroidism. She received aggressive IV fluid resuscitation, an insulin drip, electrolyte repletion, an up-titration of levothyroxine, and stress-dose corticosteroids. In the second hospitalization, she also had hypotension and electrolyte derangements, along with hypoglycemia and myxedema. She received glucose management, thyroid hormone replacement, and stress steroids again. The third hospitalization involved flu-like symptoms and a positive SARS-CoV-2 test. She was managed similarly for hypotension, hyponatremia, and hyperglycemia. In this case, she presented with non-gap metabolic acidosis and required a bicarbonate drip for a short period. She did not receive antibiotics across these three admissions.

We present three hospitalizations where adrenal, pancreatic, and thyroid derangements were seen and managed. It is known that most general providers other than endocrinologists are not comfortable with the management of disease manifestations of PAS-II; therefore, we provide a case review to address the standard of care management and guidelines with further discussion. This patient’s maintenance care was complicated by a lack of adherence to outpatient medications, leading to recurrent hospitalizations. We also endorse the importance of doctors pursuing endocrinology fellowships, especially due to the observed waning number of graduates. An endocrinologist’s availability and involvement in the care of patients with complex endocrine issues lead to improved outcomes.

## Introduction

Polyglandular autoimmune syndrome (PAS) is a rare autoimmune disorder that affects multiple endocrine organs, leading to a wide range of symptoms and complications. It is characterized by a misinterpretation of antigen recognition by the body’s immune cells, in which molecules called major histocompatibility complexes (MHC) are involved. Specific human leukocyte antigen (HLA) haplotypes such as DR3 and DR4, alleles DQ2 and DQ8, and non-HLA proteins have been associated with a predisposition to PAS-II [1].

Polyglandular autoimmune syndrome involves a spectrum of disease classifications, including PAS-I, PAS-II, PAS-III, and X-linked immune dysregulation polyendocrinopathy and enteropathy (IPEX). An iatrogenic form related to immunoregulatory agents in cancer has also been identified. Each must involve two or more endocrine organs. Type II PAS, also known as Schmidt syndrome and Carpenter syndrome, entails adrenal insufficiency, autoimmune thyroid disease, and autoimmune diabetes mellitus [1,2]. It may also present with other autoimmune manifestations, including celiac disease, alopecia, vitiligo, pernicious anemia, Stiff-man syndrome, Parkinson's disease, IgA deficiency, myasthenia gravis, pernicious anemia, herpetiformis, and hypophysitis [1].

The prevalence of PAS-II ranges from 1:1000 to 1:20,000. As with other autoimmune diseases, females are at higher risk of this genetic disorder, which usually manifests in young adulthood [1]. It is not well known why females have a greater predisposition to autoimmune disorders, but it has been postulated that there is an association with two X chromosomes [2]. Type II PAS can be difficult to diagnose and manage, and treatment often involves a multidisciplinary team approach of a primary physician, critical care, and an endocrinologist who monitor symptoms closely and prescribe thyroid and adrenal hormones as well as immunosuppressive agents. Sometimes vasopressors are needed beyond simple fluid resuscitation for shock. Examples of treatment modalities include immunosuppressants, steroids, antineoplastic agents, and hormone replacement [3]. 

We discuss a case involving three hospitalizations where adrenal shock, diabetic ketoacidosis (DKA), and myxedema were present in a patient with PAS-II. The management of this case, followed by a discussion of the guidelines, is provided for education. We also address the importance of endocrinology as a field and its involvement in the complex care of these patients.

## Case presentation

Our patient was a 21-year-old African American woman with a medical history significant for PAS-II composed of Addison’s disease, type 1 diabetes mellitus, and Hashimoto’s thyroiditis. She had other diagnoses of gastroparesis, anxiety, and chronic pulmonary embolism with chronic anticoagulation use. Her clinical course over time is detailed below.

First hospitalization

In May 2021, the patient reported traveling out of state for a month. Her primary concern was profound generalized weakness in the past week associated with an overnight episode of emesis. A chart review showed that she had four admissions for DKA in the previous year, suggesting poor disease control. The patient was able to confirm her home medications as glargine 10 units at bedtime, lispro 1:5 insulin-to-carbohydrate scale, fludrocortisone 0.1 mg daily, hydrocortisone 20 mg in the morning and 10 mg in the evening, apixaban 5 mg twice daily, metoclopramide 10 mg three times a day with meals, and escitalopram 20 mg daily. She was taking her fludrocortisone regularly but was unable to fill her hydrocortisone and levothyroxine doses routinely, despite denying any financial issues. She had received the Johnson & Johnson SARS-CoV-2 vaccine in March and had no new exposures to SARS-CoV-2. Her test was negative in the emergency department (ED).

There was no evidence of cardiopulmonary or abdominopelvic etiology on imaging, and no evidence of urinary tract infection on urinalysis. Blood cultures were obtained, but she was not started on antibiotics. An adrenal crisis was suspected based on the patient’s partial noncompliance with home medications. There was low suspicion for infection, and her blood cultures were ultimately negative for growth. An electrocardiogram (EKG) showed baseline changes of normal conduction, diffuse T-wave flattening, and inversion in precordial leads V3-V6 and limb leads I-II in a mixed distribution. She exhibited no chest pain, and her troponin-T levels were within normal range.

Due to her hypovolemic status and difficult IV access, central venous access was established in the right femoral vein and aggressive isotonic fluids were initiated for profound hypotension 70s/30s mmHg. She also met the criteria for DKA, so an insulin drip was started. Her presenting thyroid function panel entailed thyroid stimulating hormone (TSH) 247.5 mcIU/mL and free thyroxine (T4) <0.1 ng/dL. The endocrinologist recommended IV levothyroxine with a plan to transition to the oral equivalent at discharge, as well as three days of stress-dose corticosteroid to support adrenal function. Her kidney function was affected by an elevated serum creatinine of 1.4 mg/dL from a baseline of 0.67, which was treated with IV fluids. Electrolyte derangements included mild hyponatremia 130 mmol/L and moderate hyperkalemia 7.4 mmol/L which were repleted by oral and IV routes.

After stabilization of blood pressure without vasopressor support, normalization of the anion gap, and electrolyte repletion, the patient was discharged. Medication changes involved an up-titration of her levothyroxine and a three-day double-dose of her home prescription for hydrocortisone. Other home prescriptions were continued as previously prescribed.

Second hospitalization

Five months later, in October 2021, the patient presented to a stand-alone ED with shortness of breath, intractable nausea and vomiting, myalgias, and weakness for three days. Despite a known history of poor adherence to home prescriptions, she endorsed compliance but was not able to tolerate the last three days of oral medications due to profound symptoms of weakness and abdominal pain.

She was found to have significant hypotension, hypoglycemia, and electrolyte derangements, as well as myxedema and an adrenal crisis. She was not in a coma. No antibiotics had been started prior to transfer to the admitting facility. She tried food and a glucose tablet for hypoglycemia after her nausea was better controlled; however, her glucose rebounded to 500 mg/dL and she was started on an insulin drip for closer glucose monitoring and treatment. She was given IV levothyroxine and stress-dose hydrocortisone. Blood cultures obtained on admission were negative. After clinical improvement was achieved, she was stable for discharge with verbal and written instructions on hydrocortisone taper. Careful return precautions were discussed.

In 2022, she seemed to have had no hospitalizations or events needing medical evaluation, suggesting disease control. There is a paucity of information in the medical chart regarding her care during this time, so it is unclear what contributed to her better disease control. We hypothesize that she had a more stable home life or financial situation.

Third hospitalization

Nearly two years later, in January 2023, the patient presented with body aches, myalgias, nausea, and vomiting. Infectious studies were obtained, including rapid COVID/Flu/respiratory syncytial virus (RSV) detection by polymerase chain reaction. The SARS-CoV-2 ribonucleic acid was detected with a subacute cycle threshold (CT) value of 38.1. Another infectious workup was negative, so antibiotics were not started. Her blood cultures were pending but ultimately negative for growth.

**Figure 1:**
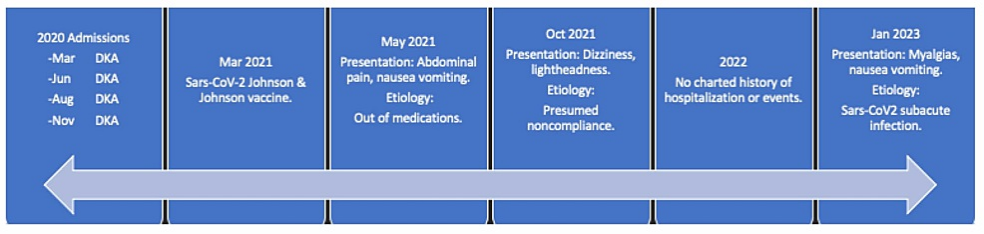
Clinical timeline DKA: Diabetic ketoacidosis

She was hypotensive as per her typical presentation, hyponatremic with a sodium level of 121 mmol/L, and hyperglycemic. The plan of care entailed isotonic fluid repletion, an insulin drip with titration, IV solumedrol, and IV levothyroxine. She continued to have difficulty with oral tolerance due to her gastric symptoms. Her venous blood gas (VBG) showed pH 7.37, partial pressure of carbon dioxide (pCO2) 29.6, partial pressure of oxygen (pO2) 106, and bicarbonate 17.2, therefore the bicarbonate drip was initiated for a mixed acid-base disorder, and non-gap metabolic acidosis. She eventually improved to tolerate oral medications for her chronic conditions over the course of her 23-day stay. On discharge, she received repeat instructions on compliance and specific verbal and written instructions for steroid taper. Medication changes are included in Table 1. Her clinical timeline is illustrated in Figure 1.

**Table 1:** Patient’s home medication list and changes during hospitalizations ac: With meals, hs: At night, I:C: Insulin-to-carbohydrate ratio, qd: Daily, bid: Twice daily, tid: Three times a day

Medication	Dose on index admission, May 2021	Changed dose, 1^st ^hospitalization	Changed dose, 2^nd^ hospitalization	Changed dose, 3^rd^ hospitalization
Glargine	10 units hs	No changes	No changes	No changes
Lispro	1:5 I:C	No changes	No changes	No changes
Hydrocortisone	20 mg AM, 10 mg PM	40 mg AM, 20 mg PM x3 days	20 mg AM, 10 mg PM	Taper: 50 mg bid x3 days, 40 mg bid x3 days, 30 mg x3 days, followed by 20 mg AM and 10 mg PM
Fludrocortisone	0.1 mg qd	No changes	No changes	No changes
Levothyroxine	250 mcg	No changes	No changes	No changes
Apixaban	5 mg bid	No changes	No changes	No changes
Metoclopramide	10 mg tid ac	No changes	No changes	No changes
Escitalopram	20 mg qd	No changes	No changes	No changes

## Discussion

The standard of care for endocrine illnesses like PAS is more well-known among endocrinologists than other providers [4]. During the patient’s first hospitalization, she did not receive empiric antibiotics for adrenal crisis or DKA. Fortunately, she did not harbor bacteremia per her negative blood cultures, but this is often unclear at presentation and infection is a common precipitant of adrenal crisis. Delaying treatment for sepsis can result in rapid decline. After a careful review of our patient's chart at the outside hospital, we noted that despite stating stress-dose steroids were ordered, these changes were not reflected in the inpatient regimen of fludrocortisone and hydrocortisone. While she was instructed to double the doses of these steroid components at discharge, it cannot be certain that verbal education was provided along with her discharge paperwork or made clear to her. Lack of proper education before patient discharge is reported to be suboptimal and the cause of about 25% of readmissions. This is a preventable cause of readmission for uncontrolled patient illnesses [5]. Zeng et al. suggest using pictographs as an aid along with discharge instructions to improve patient recall [6]. Common strategies, in addition to discharge education, known to improve outcomes include the teach-back method, follow-up calls, and meeting with a clinical pharmacist to enhance patient understanding of their disease.

It is known that our patient had poor adherence to medications from her history of frequent hospitalizations for DKA. She denied financial or other reasons why she reported difficulty obtaining her corticosteroid and thyroid management medications after travel. This may have blinded providers to the need to treat shock with empiric antibiotics. It could not be ascertained initially that she was not presenting with shock due to infection. Most cases of DKA are due to infection and often go untreated, causing delays in care that could have important clinical implications. However, to the point of antimicrobial stewardship, infection-precipitated DKA may be overreported [7].

In regard to the problem of medication adherence, a social worker’s involvement in inpatient cases is vital to ensure that patients have access to medications and the means to follow up with a provider for refills. Patients need to feel understood, believed, and respected [8]. Institutional barriers to this patient may have involved endocrinology as an underrepresented specialty, long periods of wait till an appointment, or long wait time in the office.

Despite an endocrinologist’s involvement in this patient’s care, orders were not updated to reflect recommendations, which may encourage the need for hospital systems to clarify who is responsible for updating orders. The specialist should be available for staff questions, especially in these critically ill cases. Observing the growing epidemic of endocrine disorders, the national shortage of endocrinologists should be noted. Providers interested in the specialty have declined since the 1990s and there is an increasingly predicted shortage of 2700 endocrinologists nationally by 2025 [9]. The shortage of specialists coupled with the increasing prevalence of complicated diabetic conditions is alarming. It may be of benefit to evaluate and address the confidence of hospitalists managing endocrine disorders as well as outcomes when this occurs. This may add urgency to the pressure to recruit new endocrinologists. 

The thyroid gland

**Figure 2:**
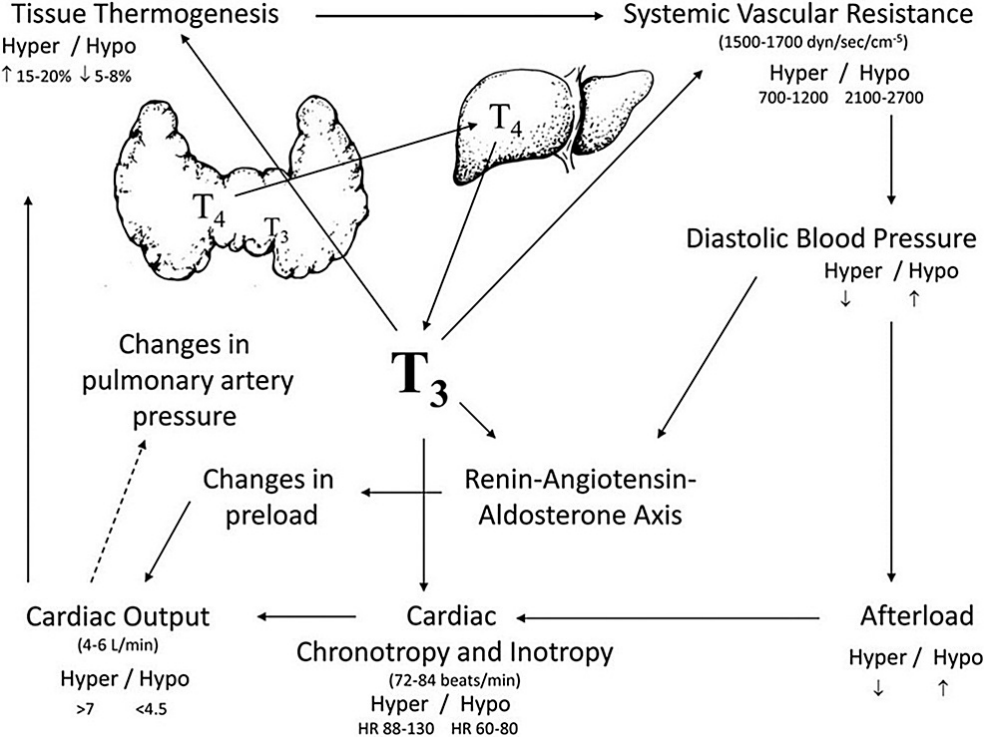
Thyroid function and effects on hemodynamics The illustration is sourced from an open-access article by Alqhatani et al. [10] and distributed under the terms of the Creative Commons Attribution License CC-BY 4.0., which permits unrestricted use, distribution, and reproduction in any medium, provided the original author and source are credited.

The thyroid regulates most body organs, including the heart (Figure 2) [10], lung, endocrine, and central nervous system functions. Signs of dysfunction may involve sleep apnea, fluid accumulation, fatigue, sexual disorder, infertility, and confusion [11]. When levels are profoundly low, patients may present with myxedema, such as in our patient’s second hospitalization, or it may progress to myxedema coma. When a thyroid crisis occurs, it can lead to serious injury and/or death, whether in a witnessed or unwitnessed environment. While the goal is to prevent such complications from occurring, in cases where death does occur, an exam and autopsy by a licensed medical forensic pathologist are warranted. The job of a forensic pathologist is to examine the evidence presented to them concerning the deceased patient to determine a cause consistent both with the information given to them and with their own findings [12].

Two main tests are used to determine thyroid function: TSH and FT4. The TSH stimulates thyroid function, but FT4 is released directly by the thyroid. The TSH levels indicate the opposite thyroid activity, i.e., elevated TSH indicates low thyroid activity and a low TSH level indicates high intrinsic thyroid activity. The FT4 levels are synonymous with the direction of thyroid activity: low FT4 indicates low activity and high FT4 indicates high intrinsic thyroid activity [13].

The thyroid is the end target of the hypothalamus-pituitary-thyroid axis. As such, thyroid hormones normally inhibit the pituitary release of TSH through feedback inhibition. Loss of inhibition leads to excess TSH, resulting in hypothyroidism. In hyperthyroid states, excess T4 causes a greater-than-normal decrease in TSH [13]. The reason for obtaining TSH levels is related to cost. The T4 tests are more expensive; therefore, TSH is generally used diagnostically while T4 is used for monitoring disease activity [13].

The adrenal gland

The adrenal gland is a humble, profoundly vital organ where dysfunction leads to varying levels of discomfort, rapid decline including shock, and death if not promptly treated. It is located above the kidney with a blood supply from the superior adrenal arteries, branches of the inferior phrenic artery, the middle adrenal artery, and the inferior adrenal artery [14]. It is divided into the cortex, the outer level that produces hormones, and the medulla, which produces catecholamines, epinephrine, and norepinephrine. The cortex is divided into the outermost zona glomerulosa, which produces mineralocorticoids; the middle zona fasciculata, which produces glucocorticoids; and the innermost zona reticularis, which produces androgens. These regions and their production are supplemented by adrenal insufficiency (Table 2). Mineralocorticoids, such as aldosterone, regulate the balance of water via sodium and potassium concentrations. Deficiency can lead to muscle weakness, arrhythmias, and hypotension. Glucocorticoids such as cortisol have a wide variety of effects on the body, including stress response, regulation of metabolism, immunity, and blood pressure regulation. As a result, glucocorticoid deficiency causes a wide variety of symptoms, including fatigue, depression/anxiety, weakened immunity, and orthostatic hypotension. Androgens such as dehydroepiandrosterone and testosterone are important for proper sexual development and function as well as for maintaining energy levels. In females, the adrenals are the main source of androgens, and as such, deficiency can cause fatigue and loss of sexual interest. Males are comparatively spared from adrenal androgen deficiency given that gonads are their main source of androgens [15].

**Table 2:** Classes of drugs to treat manifestations of PAS-II DMARD: Disease-modifying antirheumatic drug, N/A: Not applicable, PAS-II: Polyglandular autoimmune syndrome type II

Drug class	Common names	Natural production
Glucocorticoid	Prednisone, prednisolone, methylprednisolone, dexamethasone, triamcinolone	Adrenal cortex, zona fasciculata
Corticosteroid	Fludrocortisone, hydrocortisone	Adrenal cortex, zona glomerulosa
Calcineurin inhibitor	Sirolimus, tacrolimus	N/A
Antineoplastic agent/DMARD	Methotrexate, mycophenolate mofetil, rituximab	N/A

Guideline-directed management published by the Endocrine Society task force for adrenal insufficiency and adrenal shock, begins with a blood test to measure adrenocorticotrophic hormone (ACTH) and establish a diagnosis. Simultaneously, levels of renin and aldosterone should be measured to determine if hormones other than glucocorticoids are affected. In a patient with confirmed adrenal insufficiency presenting with shock, these tests should be obtained but should not delay treatment. Therapy consists of glucocorticoid replacement, with doubling or tripling the maintenance dose for three days alongside fluid resuscitation and electrolyte repletion. If the patient also has a confirmed aldosterone deficiency, fludrocortisone replacement should be initiated [16].

When major surgery, trauma, delivery, or disease requires intensive care needs, hydrocortisone should be given at a dose of 100 mg IV followed by an IV infusion of 200 mg per 24 hours, or 50 mg every six hours with IV and intramuscular (IM) equivalency. Children require weight-based dosing. Maintenance fluids containing dextrose should also be given. In an acute adrenal crisis, saline resuscitation should be prioritized, followed by maintenance fluids, and hydrocortisone should be given as above, i.e., 100 mg IV followed by 200 mg over 24 hours. Children, again, require weight-based dosing of 50 to 100 mg/m2/day [16]. If an infection is at all suspected, antibiotics should be added, which was the discrepancy in our case where antibiotics were not administered during hospitalizations.

Patients should be given an emergency steroid pack and educated on the sick day rule for doubling the maintenance hydrocortisone dose on sick day 1. On sick day 2, injectables should be started. On sick day 3, injectable steroids should be scheduled and administered in a medical setting under close observation. Patients should wear an adrenal insufficiency alert bracelet that states the need for steroids and medical care. Patients should also be provided with the emergency number of an endocrinologist [16].

## Conclusions

Polyglandular autoimmune syndrome type II can be difficult to diagnose and treat. We discussed the anatomy and physiology of the thyroid and adrenal glands to address the gap in understanding and provide a series of three hospital presentations in a young African American woman with Addison’s disease, hypothyroidism, and insulin-dependent diabetes mellitus. Her presentation during uncontrolled periods of disease was with varying degrees of adrenal crisis, myxedema, and glucose and electrolyte derangements, but she did have gap years of good disease control. We encourage the recruitment of more diverse endocrinologists to meet the needs of the population. While we encourage the involvement of an endocrinologist when available, the specialty is known to be experiencing a shortage, and our article provides a brief overview to improve provider recall of the disease and priorities of treatment.
